# Supplementary material for: Targeting glutamine metabolism improves sarcoma response to radiation therapy in vivo
Source: Commun Biol. 2024 May 20;7:608. doi: 10.1038/s42003-024-06262-x (PMC11106276; doi:10.1038/s42003-024-06262-x)
Supplement: Supplementary file 2 — Supplementary Information [file 42003_2024_6262_MOESM2_ESM.pdf]

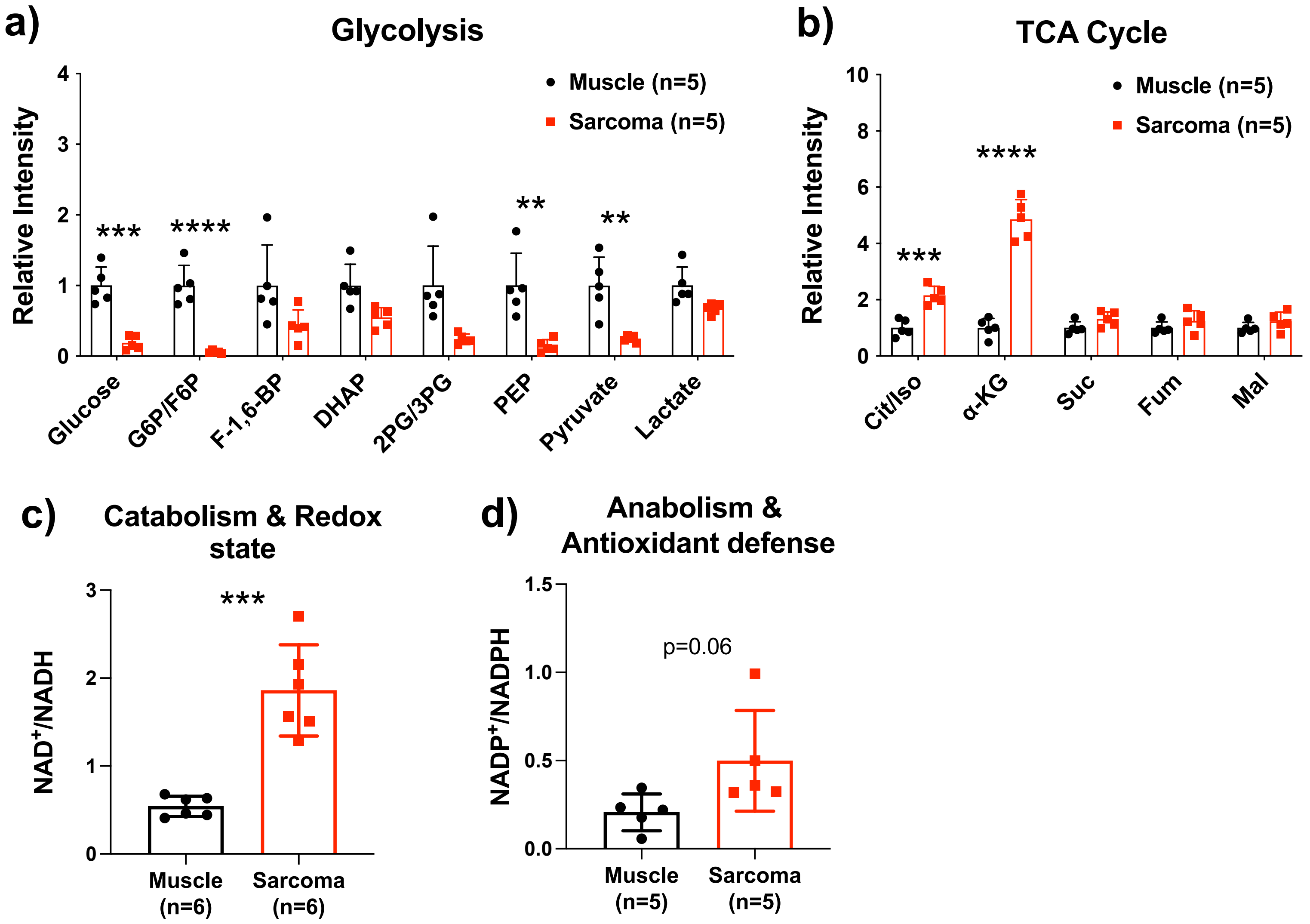

Supplementary figure 1: Differentially regulated metabolites in normal muscle versus sarcoma. Scatter bar graphs showed metabolite abundance in (a) Glycolysis and (b) TCA cycle pathways. p-values were calculated using multiple t-tests. Comparison of the redox and antioxidant defense state of normal muscle versus sarcoma. The ratio of (c) $NAD^+/NADH$ highlighting catabolism and redox state, and (d) $NADP^+/NADPH$ highlighting anabolism and antioxidant defense state of normal muscle versus sarcoma. p-values were calculated using t-tests. All data are represented as means ± SD; *** $p < 0.001$; n = number of tissue samples.

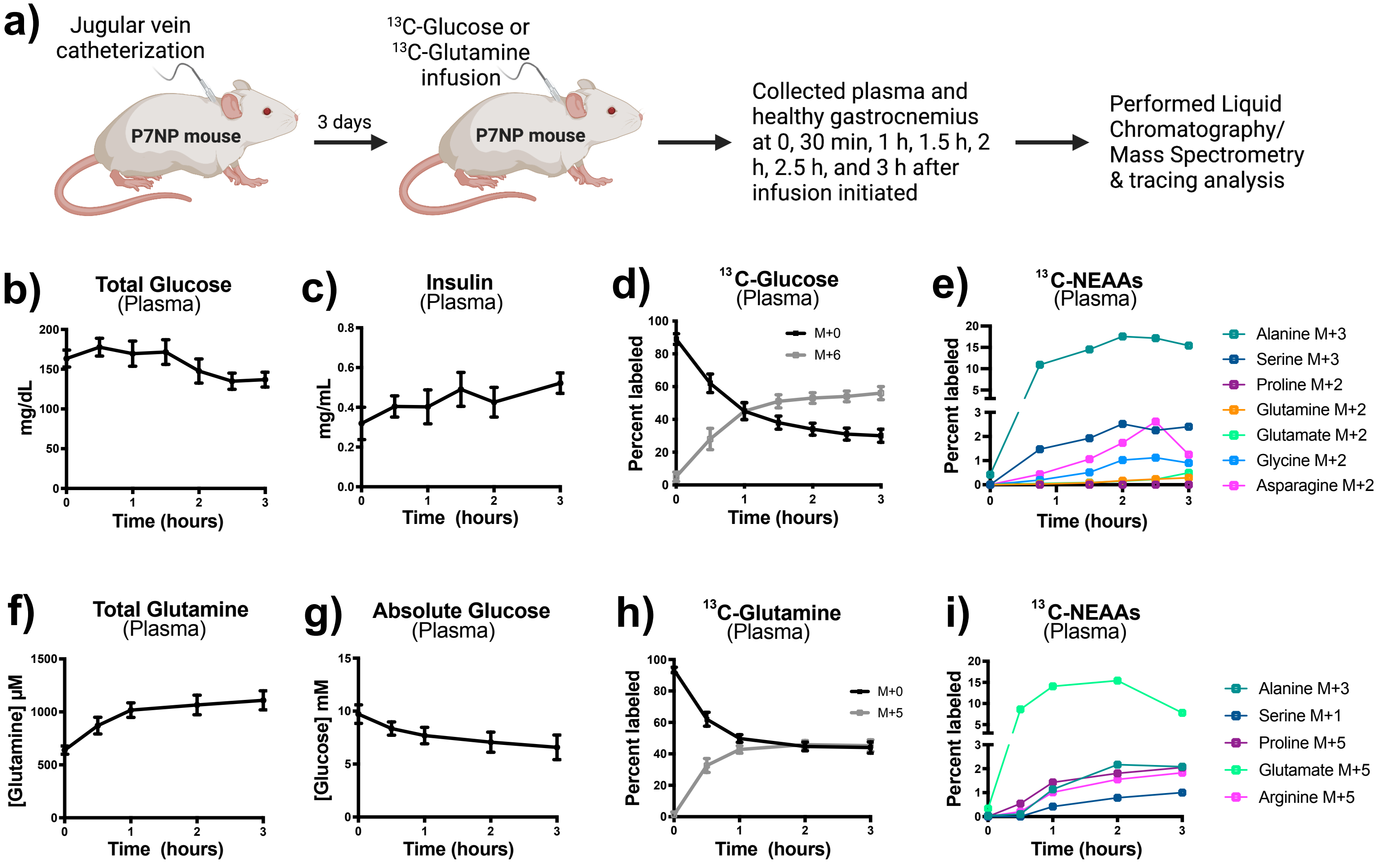

Supplementary figure 2: Euglycemic glucose and glutamine infusions label non-essential amino acids in plasma. (a) Schematic of [U-^{13}C]glucose and [U-^{13}C]glutamine infusions in healthy C57BL/6 mice and LC/MS analyses. (b) Plasma glucose throughout ^{13}C-glucose infusion (n=7). (c) Plasma insulin throughout ^{13}C-glucose infusion (n=7). (d) ^{13}C-glucose enrichment in plasma over time. M+0 unlabeled, M+6 fully labeled (n=7). (e) ^{13}C-labeled non-essential amino acids (NEAAs) in plasma over time (n=7). (f) Total plasma glutamine throughout ^{13}C-glutamine infusion (n=5). (g) Plasma glucose throughout ^{13}C-glutamine infusion (n=5).(h) ^{13}C-glutamine enrichment in plasma over time. M+0 unlabeled, M+5 fully labeled (n=5). (i) ^{13}C-labeled non-essential amino acids in plasma of glutamine-infused mice (n=5). n = number of mice.

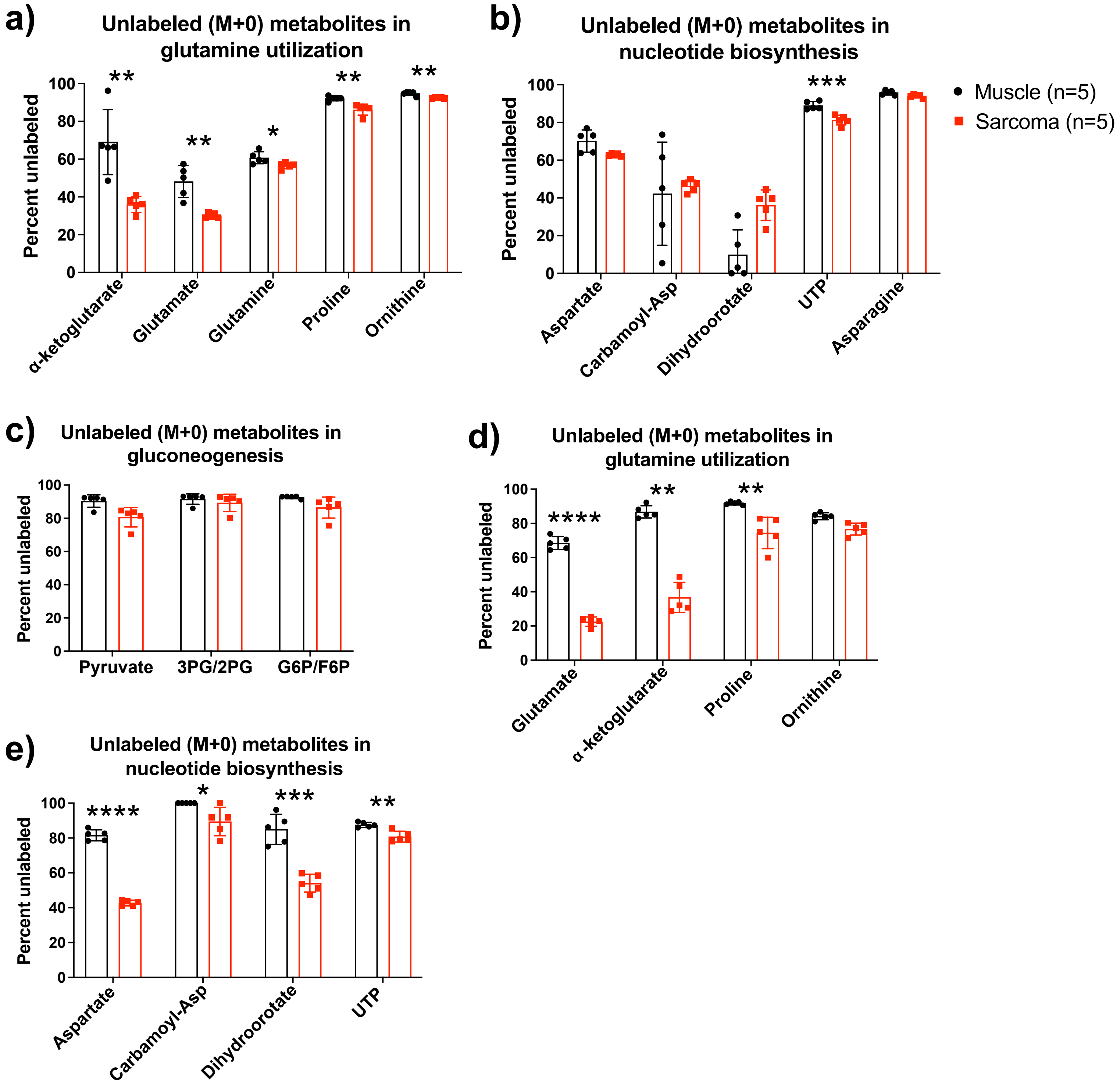

Supplementary figure 3: Unlabeled (M+0) metabolites abundance in different metabolic pathways. Percentage unlabeled (M+0) metabolites involved in (a) Glutamine utilization pathway, and (b) Nucleotide biosynthesis pathway during [U-^{13}C]glucose infusion in P7NP sarcoma-bearing mice (n=5). Percentage unlabeled (M+0) metabolites involved in (c) Gluconeogenesis pathway, (d) Glutamine utilization pathway, and (e) Nucleotide biosynthesis pathway during [U-^{13}C]glutamine infusion in P7NP sarcoma-bearing mice (n=5). p-values in scatter bar graphs were calculated using multiple t-tests. All data were presented as means ± S.D. * $p < 0.05$, ** $p < 0.01$, *** $p < 0.001$, **** $p < 0.0001$. n = number of mice.

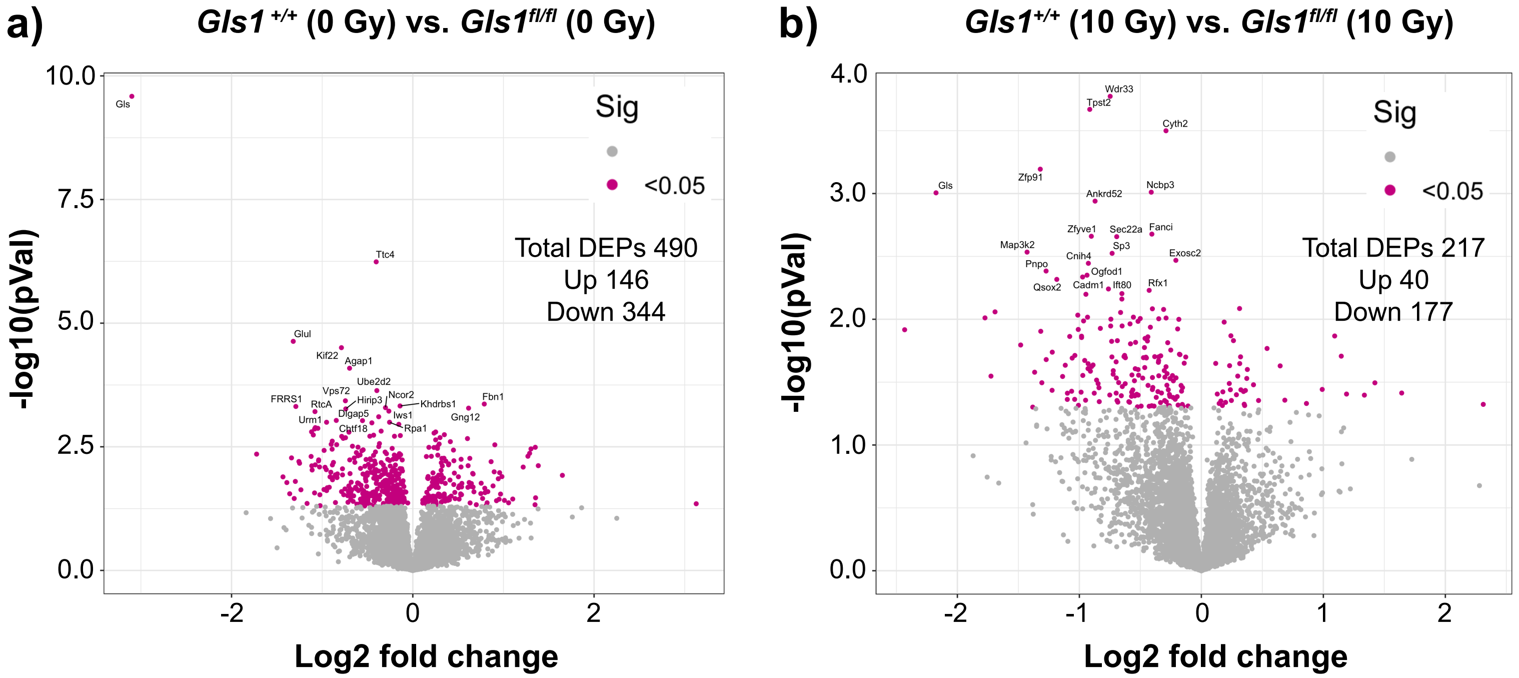

Supplementary figure 4: Unlabeled proteomic analysis showed the impact of *Gls1* deletion and/or RT on protein expression in sarcomas. Volcano plots illustrated differentially expressed proteins (DEPs) in (a) *Gls1*$^{+/+}$ versus *Gls1*$^{fl/fl}$ without RT, and (b) *Gls1*$^{+/+}$ versus *Gls1*$^{fl/fl}$ post-RT. p-values cut offs were set at an adjusted p-value ≤ 0.05. No fold-change cut-off was used.

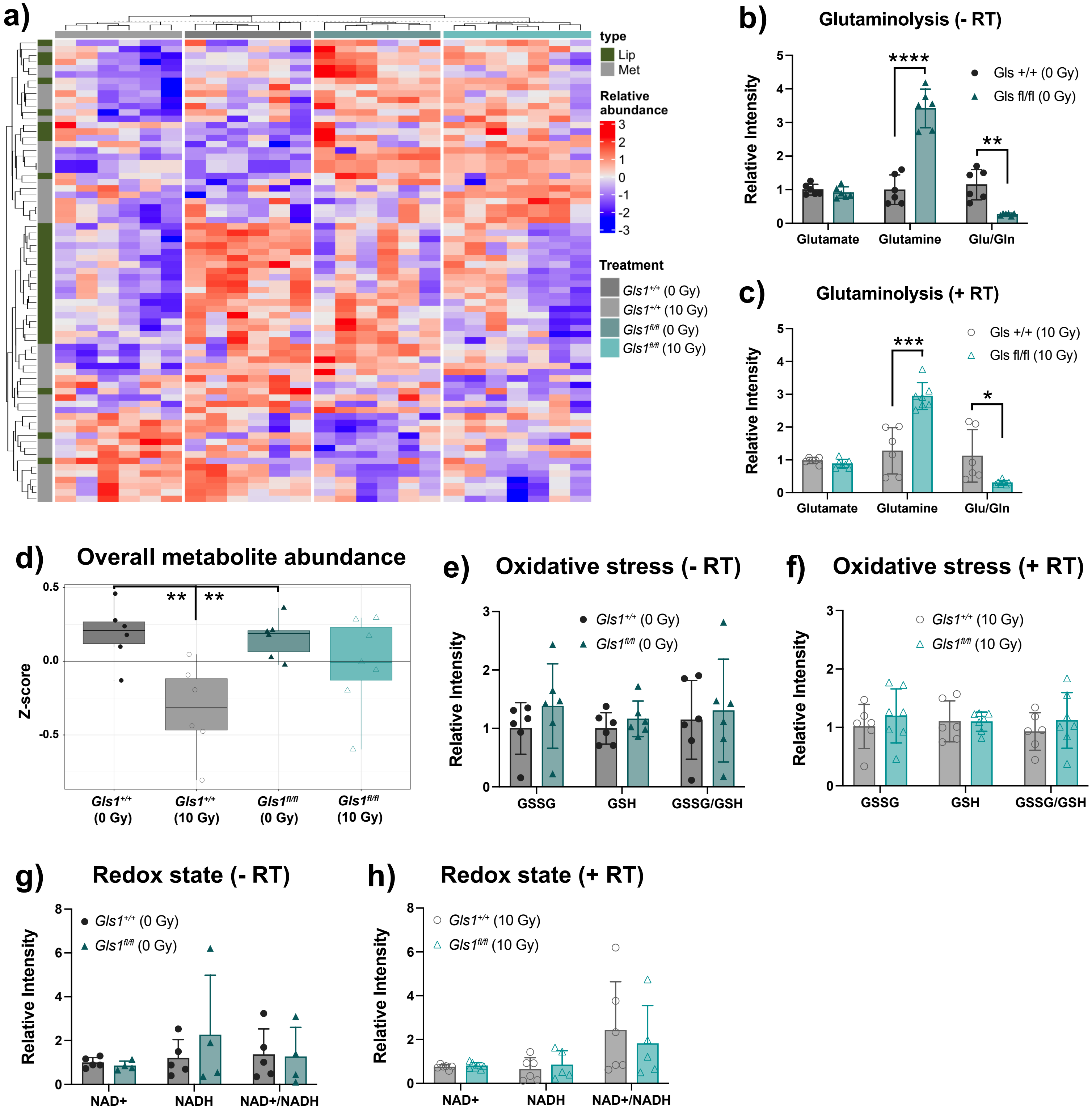

Supplementary figure 5: Unlabeled metabolomic analysis showed the impact of *Gls1* deletion and/or RT on metabolite abundance in sarcomas. (a) Heatmap showed differentially abundant metabolites and lipids across all treatment groups. Scatter bar plots showed the amount of glutaminolysis in (b) *Gls1*$^{+/+}$ versus *Gls1*$^{fl/fl}$ P7NP sarcomas without RT, and (c) *Gls1*$^{+/+}$ versus *Gls1*$^{fl/fl}$ P7NP sarcomas post-RT. (d) Box & whiskers plot showed z-scores comparing the relative abundance of the differentially abundent metabolites (polar and non-polar). Scatter bar graphs showed oxidative stress (e) without RT and (f) post-RT in *Gls1*$^{+/+}$ and *Gls1*$^{fl/fl}$ sarcomas. Scatter bar graphs showed redox state (g) without RT and (h) post-RT in *Gls1*$^{+/+}$ and *Gls1*$^{fl/fl}$ sarcomas. Associated p-values comparing z-scores across treatment groups was calculated using Wilcoxon test. p-values in scatter bar graphs were calculated using multiple t-tests. All data are presented as means ± SD; * $p < 0.05$, ** $p < 0.01$, *** $p < 0.001$, **** $p < 0.0001$; RT - radiation therapy.
