# Supplementary material for: Targeting glutamine metabolism improves sarcoma response to radiation therapy in vivo
Source: Commun Biol. 2024 May 20;7:608. doi: 10.1038/s42003-024-06262-x (PMC11106276; doi:10.1038/s42003-024-06262-x)
Supplement: Supplementary file 3 — Description of Additional Supplementary Materials [file 42003_2024_6262_MOESM3_ESM.docx]

**Description of Additional Supplementary Files**

**File name:** Supplementary Data

**Description:** all numerical source data used to generate each figure
