# Supplementary material for: Targeting glutamine metabolism improves sarcoma response to radiation therapy in vivo
Source: Commun Biol. 2024 May 20;7:608. doi: 10.1038/s42003-024-06262-x (PMC11106276; doi:10.1038/s42003-024-06262-x)
Supplement: Supplementary file 5 — Reporting Summary [file 42003_2024_6262_MOESM5_ESM.pdf]

nature portfolio

Corresponding author(s): David G. Kirsch

Last updated by author(s): Mar 17, 2024

Reporting Summary

Nature Portfolio wishes to improve the reproducibility of the work that we publish. This form provides structure for consistency and transparency in reporting. For further information on Nature Portfolio policies, see our Editorial Policies and the Editorial Policy Checklist.

Statistics

For all statistical analyses, confirm that the following items are present in the figure legend, table legend, main text, or Methods section.

n/a	Confirmed	
☐	☒	The exact sample size (*n*) for each experimental group/condition, given as a discrete number and unit of measurement
☐	☒	A statement on whether measurements were taken from distinct samples or whether the same sample was measured repeatedly
☐	☒	The statistical test(s) used AND whether they are one- or two-sided<br>*Only common tests should be described solely by name; describe more complex techniques in the Methods section.*
☐	☒	A description of all covariates tested
☐	☒	A description of any assumptions or corrections, such as tests of normality and adjustment for multiple comparisons
☐	☒	A full description of the statistical parameters including central tendency (e.g. means) or other basic estimates (e.g. regression coefficient) AND variation (e.g. standard deviation) or associated estimates of uncertainty (e.g. confidence intervals)
☐	☒	For null hypothesis testing, the test statistic (e.g. *F*, *t*, *r*) with confidence intervals, effect sizes, degrees of freedom and *P* value noted<br>*Give P values as exact values whenever suitable.*
☐	☒	For Bayesian analysis, information on the choice of priors and Markov chain Monte Carlo settings
☐	☒	For hierarchical and complex designs, identification of the appropriate level for tests and full reporting of outcomes
☐	☒	Estimates of effect sizes (e.g. Cohen's *d*, Pearson's *r*), indicating how they were calculated

Our web collection on statistics for biologists contains articles on many of the points above.

Software and code

Policy information about availability of computer code

Data collection	N/A
Data analysis	N/A

For manuscripts utilizing custom algorithms or software that are central to the research but not yet described in published literature, software must be made available to editors and reviewers. We strongly encourage code deposition in a community repository (e.g. GitHub). See the Nature Portfolio guidelines for submitting code & software for further information.

Data

Policy information about availability of data

All manuscripts must include a data availability statement. This statement should provide the following information, where applicable:

- Accession codes, unique identifiers, or web links for publicly available datasets
- A description of any restrictions on data availability
- For clinical datasets or third party data, please ensure that the statement adheres to our policy

The mass spectrometry proteomics data have been deposited to the ProteomeXchange Consortium via the PRIDE partner repository with the dataset identifier PXD050936.

Research involving human participants, their data, or biological material

Policy information about studies with human participants or human data. See also policy information about sex, gender (identity/presentation), and sexual orientation and race, ethnicity and racism.

Reporting on sex and gender	N/A
Reporting on race, ethnicity, or other socially relevant groupings	N/A
Population characteristics	N/A
Recruitment	N/A
Ethics oversight	N/A

Note that full information on the approval of the study protocol must also be provided in the manuscript.

Field-specific reporting

Please select the one below that is the best fit for your research. If you are not sure, read the appropriate sections before making your selection.

☒ Life sciences ☐ Behavioural & social sciences ☐ Ecological, evolutionary & environmental sciences

For a reference copy of the document with all sections, see nature.com/documents/nr-reporting-summary-flat.pdf

Life sciences study design

All studies must disclose on these points even when the disclosure is negative.

Sample size	Power and sample size for animal survival studies were calculated to be 20 mice/group which provided 80% statistical power to detect differences between groups.
Data exclusions	No data or mice were excluded from the study.
Replication	Animal studies were not replicated. However, animal number utilized for each experiment were provided in figure, figure legends, and method sections.
Randomization	Animals were randomized before radiation therapy and/or drug treatment.
Blinding	Animal groups were kept blinded from the observers.

Reporting for specific materials, systems and methods

We require information from authors about some types of materials, experimental systems and methods used in many studies. Here, indicate whether each material, system or method listed is relevant to your study. If you are not sure if a list item applies to your research, read the appropriate section before selecting a response.

Materials & experimental systems

n/a	Involved in the study	
☐	☒	Antibodies
☐	☐	Eukaryotic cell lines
☐	☐	Palaeontology and archaeology
☐	☒	Animals and other organisms
☐	☐	Clinical data
☐	☐	Dual use research of concern
☐	☐	Plants

Methods

n/a	Involved in the study	
☐	☐	ChIP-seq
☐	☒	Flow cytometry
☐	☐	MRI-based neuroimaging

Antibodies

Antibodies used	The following antibodies were utilized for flow cytometry analysis.<br>APC-Cy7 conjugated anti-mouse CD45 (BD biosciences, Cat # 557659), BV711 conjugated anti-mouse CD3 (BioLegend, Cat # 100241),

	PE conjugated anti-mouse NKp46 (BD biosciences, Cat # 560757), AF488 conjugated anti-mouse CD11c (BioLegend, Cat # 117313), and BV421 conjugated anti-mouse CD11b (BioLegend, Cat # 101235)
Validation	All antibodies were validated by manufacturers. We did not validated antibodies in our laboratory.

Eukaryotic cell lines

Policy information about cell lines and Sex and Gender in Research

Cell line source(s)	N/A
Authentication	N/A
Mycoplasma contamination	N/A
Commonly misidentified lines (See ICLAC register)	N/A

Palaeontology and Archaeology

Specimen provenance	N/A
Specimen deposition	N/A
Dating methods	N/A

☐ Tick this box to confirm that the raw and calibrated dates are available in the paper or in Supplementary Information.

Ethics oversight	N/A

Note that full information on the approval of the study protocol must also be provided in the manuscript.

Animals and other research organisms

Policy information about studies involving animals; ARRIVE guidelines recommended for reporting animal research, and Sex and Gender in Research

Laboratory animals	All animal studies were performed using Mus Musculus. Animals were mixed-strains (breed between C57BL/6 and 129/SvJae strains). For experiments, 6-12 weeks old mice were utilized to generate primary tumors and mice were followed up to the study endpoint.
Wild animals	N/A
Reporting on sex	Male and female mice were utilized for all animal experiments at the ratio of 1:1
Field-collected samples	N/A
Ethics oversight	All animal procedures were approved by the Institutional Animal Care and Use Committee (IACUC) at Duke University.

Note that full information on the approval of the study protocol must also be provided in the manuscript.

Clinical data

Policy information about clinical studies

All manuscripts should comply with the ICMJE guidelines for publication of clinical research and a completed CONSORT checklist must be included with all submissions.

Clinical trial registration	N/A
Study protocol	N/A
Data collection	N/A
Outcomes	N/A

Dual use research of concern

Policy information about dual use research of concern

Hazards

Could the accidental, deliberate or reckless misuse of agents or technologies generated in the work, or the application of information presented in the manuscript, pose a threat to:

No	Yes	
☒	☐	Public health
☒	☐	National security
☒	☐	Crops and/or livestock
☒	☐	Ecosystems
☒	☐	Any other significant area

Experiments of concern

Does the work involve any of these experiments of concern:

No	Yes	
☒	☐	Demonstrate how to render a vaccine ineffective
☒	☐	Confer resistance to therapeutically useful antibiotics or antiviral agents
☒	☐	Enhance the virulence of a pathogen or render a nonpathogen virulent
☒	☐	Increase transmissibility of a pathogen
☒	☐	Alter the host range of a pathogen
☒	☐	Enable evasion of diagnostic/detection modalities
☒	☐	Enable the weaponization of a biological agent or toxin
☒	☐	Any other potentially harmful combination of experiments and agents

Plants

Seed stocks	N/A
Novel plant genotypes	N/A
Authentication	N/A

ChIP-seq

Data deposition

☐ Confirm that both raw and final processed data have been deposited in a public database such as GEO.

☐ Confirm that you have deposited or provided access to graph files (e.g. BED files) for the called peaks.

Data access links<br>*May remain private before publication.*	N/A
Files in database submission	N/A
Genome browser session<br>(e.g. UCSC)	N/A

Methodology

Replicates	N/A

Sequencing depth	N/A
Antibodies	N/A
Peak calling parameters	N/A
Data quality	N/A
Software	N/A

Flow Cytometry

Plots

Confirm that:

- [x] The axis labels state the marker and fluorochrome used (e.g. CD4-FITC).
- [x] The axis scales are clearly visible. Include numbers along axes only for bottom left plot of group (a 'group' is an analysis of identical markers).
- [ ] All plots are contour plots with outliers or pseudocolor plots.
- [x] A numerical value for number of cells or percentage (with statistics) is provided.

Methodology

Sample preparation	Tumors were minced and digested using a tumor dissociation kit (Miltenyi Biotec, Cat # 130-096-730) for 45 minutes on a shaker at 37C. Dissociated tumor cells were strained through a 70 micron filter and washed with FACS buffer containing HBSS (Thermofisher, Cat # 14175095), 0.5 mM EDTA (Sigma-Aldrich, Cat # E7889-100ML), and 2.5% FBS (Gibco, Cat # 16000044). Red blood cells were lysed using ACK lysis buffer (Lonza, Cat # 10-548E) and washed with FACS buffer. Finally, the total number of cells was counted using a hemocytometer, and 2.5M cells/tumor were used for antibody staining.<br>Tumor cells were blocked with 1% purified rat anti-mouse CD16/CD32 (BD biosciences, Cat # 553142) for 15 minutes at room temperature before staining with cell surface antibodies. After blocking, tumor cells were stained with APC-Cy7 conjugated anti-mouse CD45 (BD biosciences, Cat # 557659), BV711 conjugated anti-mouse CD3 (BioLegend, Cat # 100241), PE conjugated anti-mouse NKp46 (BD biosciences, Cat # 560757), AF488 conjugated anti-mouse CD11c (BioLegend, Cat # 117313), and BV421 conjugated anti-mouse CD11b (BioLegend, Cat # 101235). Next, tumor cells were stained with Live/Dead Zombie Aqua fixable dye (BioLegend, Cat # 423101). All antibodies were used as per the company's recommendation
Instrument	BD LSRII cell analyzer
Software	Flow cytometry data were analyzed using FlowJo software.
Cell population abundance	Data were collected from roughly 400,000 cells/events (each tumor sample).
Gating strategy	During the data analysis, dead cells were first excluded using the viability dye Zombie Aqua, and then the single-cell population was isolated by plotting FSC-H vs. FSC-A. Immune cells were identified by plotting SSC-A vs. APC-Cy7-A (CD45+). After the identification of immune cells, two-parameter density plots were utilized to identify NK cells (NKp46-A vs. BV711-A), dendritic cells (AF488-A vs. BV711-A), and myeloid cells (BV412-A vs. BV711-A).

- [x] Tick this box to confirm that a figure exemplifying the gating strategy is provided in the Supplementary Information.

Magnetic resonance imaging

Experimental design

Design type	N/A
Design specifications	N/A
Behavioral performance measures	N/A

Acquisition

Imaging type(s)	N/A
Field strength	N/A
Sequence & imaging parameters	N/A
Area of acquisition	N/A

Diffusion MRI ☐ Used ☒ Not used

Preprocessing

Preprocessing software	N/A
Normalization	N/A
Normalization template	N/A
Noise and artifact removal	N/A
Volume censoring	N/A

Statistical modeling & inference

Model type and settings	N/A
Effect(s) tested	N/A

Specify type of analysis: ☐ Whole brain ☐ ROI-based ☐ Both

Statistic type for inference (See Eklund et al. 2016)	N/A
Correction	N/A

Models & analysis

n/a	Involved in the study
☒	☐ Functional and/or effective connectivity
☒	☐ Graph analysis
☒	☐ Multivariate modeling or predictive analysis
